# Optical Properties of Plasma Dimer Nanoparticles for Solar Energy Absorption

**DOI:** 10.3390/nano11102722

**Published:** 2021-10-15

**Authors:** Chunlei Sun, Caiyan Qin, Han Zhai, Bin Zhang, Xiaohu Wu

**Affiliations:** 1 College of Electromechanical Engineering, Qingdao University of Science and Technology, Qingdao 266061, China; 2020030062@mails.qust.edu.cn; 2 Applied Science Research Institute, Korea Advanced Institute of Science and Technology, Daejeon 34141, Korea; caiyanhit@kaist.ac.kr; 3 Shandong Institute of Advanced Technology, Jinan 250100, China; zhaihan@njust.edu.cn

**Keywords:** nanofluids, photothermal conversion, dimer, localized surface plasmon resonance

## Abstract

Plasmonic nanofluids have excellent optical properties in solar energy absorption and have been widely studied in solar thermal conversion technology. The absorption of the visible region of solar energy by ordinary metal nanoparticles is usually limited to a narrow resonance band, so it is necessary to enhance the coupling effect of nanoparticles in the visible spectrum region to improve absorption efficiency. However, it is still a difficult task to improve solar energy absorption by adjusting the structure and performance of nanoparticles. In this paper, a plasma dimer Ag nanoparticle is proposed to excite localized surface plasmon resonance (LSPR). Compared with an ordinary Ag nanoparticle in the visible region, the plasmonic Ag dimer nanoparticle produces more absorption peaks and broader absorption bands, which can broaden solar energy absorption. By analyzing the electromagnetic field of the nanoparticle, the resonance mode of the plasma dimer is discussed. The effects of the geometric dimensions of the nanoparticle and the embedding of two spheres on the optical properties are studied. In addition, the effects of a trimer and its special structure on the optical properties are also analyzed. The results show that the proposed plasma dimer Ag nanoparticle has broad prospects for application in solar thermal conversion technology.

## 1. Introduction

With the proposal of carbon neutrality in the Paris Climate Change Agreement signed by nearly 200 countries in 2016, the strategic position of renewable energy represented by solar energy and wind energy has gradually become prominent [1,2]. As a new type of energy in line with the present development trend, solar energy has attracted the attention of an increasing number of researchers due to its advantages of having huge storage and wide distribution and being completely clean [3]. Solar energy has potential for applications of photothermal conversion [4,5], solar power generation [6,7], photochemistry [8] and photobiology [9]. For example, photothermal conversion requires that devices concentrate solar radiation energy and convert it into a sufficiently high temperature to meet the needs of different loads. Photothermal conversion technology is widely used in solar collectors [10,11]. The working principle of solar collectors is to generate heat energy by absorbing solar radiation and then to transfer it to the working medium. The absorption of solar energy is the most important process in the application of a solar collector. Therefore, improving the absorption efficiency of a working medium to solar energy, one of the top ten scientific problems of human social development in 2020 [12], is the key to optimizing photothermal conversion in solar energy utilization.

Since solar energy is widely distributed across the Earth’s surface, solar energy density is relatively low. Thus, it is important to concentrate solar energy as much as possible to match the huge energy requirements. A solar collector, as a device that collects and converts the radiant energy of the sun into heat energy, makes it possible to solve this problem. However, the familiar working fluids in solar collectors, such as water [13,14], cannot meet the ideal requirements due to their limited absorption capacity. Therefore, to improve the absorption efficiency of solar energy, changing the working fluid of the solar collector is required.

Nanofluids [15,16,17,18] are produced by suspending nanoparticles (NPs) (particles less than 100 nm in size) in a base fluid (such as water). Compared with the traditional base solution, nanofluids can obtain higher heat transfer properties [19,20], which is of great significance for the absorption of solar energy. At present, the application of nanofluids in a direct absorption solar collector (DASC) can significantly enhance the heat transfer and solar energy absorption capacity and greatly improve the performance of DASC [21,22,23]. Researchers have invested in a lot of studies on the application of nanofluids in solar collectors [24,25,26,27,28]. Due to the interaction between light and nanoparticles, local surface plasmon resonance (LSPR) [29] can be excited in nanoparticles. The incident solar radiation matches the overall vibration frequency of metal nanoparticles, and nanoparticles will strongly absorb the solar radiation. At this time, a strong resonance absorption peak will appear in the spectrum, resulting in a high photothermal conversion efficiency [30,31]. However, the absorption of the visible region of solar energy by ordinary metal nanoparticles is usually limited to a narrow resonance band [32,33,34]. Therefore, in order to enhance the absorption of solar energy, researchers have a strong interest in the exploration of nanoparticles. Chen [35] proposed a plasma dimer Au nanoparticle composed of spheres and rods to improve the absorption efficiency of solar energy and achieve broadband absorption. The underlying mechanism was found to be the photonic superposition of local surface plasmon resonance, propagating surface plasmon resonance, and gap resonances at different wavelengths. The solar energy absorption efficiency of dimer nanofluids is 21.2% higher than that of mixed nanofluids composed of an equal number of spheres and rods, indicating that plasma dimer nanoparticles have a better solar energy absorption capacity, which is conducive to the broadband absorption of solar energy.

In order to enhance the coupling effect of nanoparticles in the visible light region and improve their absorption efficiency, a plasma dimer Ag nanoparticle composed of spheres of the same size is proposed in this work. There are many ways to predict and understand the optical response of nanoparticles [36]. In this work, the absorption and scattering efficiencies of particles are calculated by the finite element method (FEM) [37], and the resonance mode is analyzed by the obtained electromagnetic field under resonance conditions. Compared with ordinary spherical nanoparticles, plasmonic dimer nanoparticles are excited with more absorption peaks, which can broaden solar energy absorption. Through the analysis of the electromagnetic fields of the particle, the resonance mode of the plasma dimer is discussed. Moreover, the effects of the size parameters of the nanoparticle and the embedding of two spheres on the optical properties are studied. The effects of a trimer and its special structures on the optical properties are also studied.

## 2. Modeling

**Figure 1:**
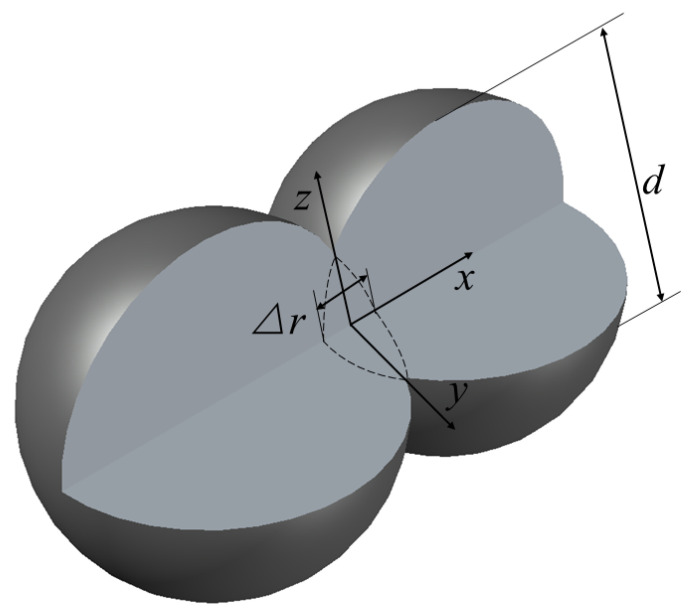
The structure diagram of the proposed Ag plasma dimer nanoparticle.

Figure 1 shows the structure of the proposed plasma dimer Ag nanoparticle composed of two Ag spheres of the same size. The purpose of choosing Ag as the object in this work is that Ag has a smaller loss factor than other metals [38]. The structure, dimension and dielectric properties of nanoparticles have an important impact on the absorption characteristics of nanoparticles [39,40,41]. The diameter of the Ag sphere is set to *d* = 30 nm. In synthesis engineering, it is difficult for two Ag spheres to be completely tangent, so the case of particle embedding is considered here. Δ*r* is the embedding depth, and the default setting is 1 nm. The surrounding working fluid is water. These dimensions are fixed in this study until further explanation. The optical constants of Ag and water are obtained from Reference [42]. In this study, the spectrum between 300 nm and 1100 nm is mainly considered. The wave optical module in COMSOL Multiphysics 5.5 is used to calculate the absorption and scattering efficiencies of the nanoparticle using the finite element method, and then the electric and magnetic fields of the particles at the resonance wavelengths are analyzed. In addition, we use the resistive heat of the nanoparticles at the resonance peak to further illustrate the influence of the plasma nanoparticles on the photothermal conversion of solar collectors.

The optical response of nanoparticles is characterized by the two dimensionless quantities, scattering efficiency *Q_sca_* and absorption efficiency *Q_abs_* [43]. Moreover, the scattering and absorption efficiency of the nanoparticle can be given by [44]

(1) $$\sigma_{sca}=\frac{1}{I_{0}}\iint \left(n\cdot S_{sca}\right)dS$$

(2) $$\sigma_{abs}=\frac{1}{I_{0}}∭QdV$$

(3) $$Q_{sca}=\sigma_{sca}/\sigma$$

(4) $$Q_{abs}=\sigma_{abs}/\sigma$$

where *σ_sca_* and *σ_abs_* are the absorption and scattering cross-sections, which represent the ratio of total scattering and absorption intensity to incident light intensity, respectively; *I*_0_ is the intensity of the incident light; ***n*** is the normal vector; ***S**_sca_* is the scattered intensity vector; *Q* is the power loss density; *S* is the surface area of the nanoparticle; *V* is the volume of the nanoparticle; and *σ* is the projected area of the nanoparticle.

Moreover, the heat power density is calculated by [45]

(5) $$Q_{rh}=\frac{1}{2}\varepsilon_{0}\omega \varepsilon^{"}{\left|E\right|}^{2}$$

where *ε*_0_ is the vacuum permittivity, *ω* is the frequency of the incident light, *ε*’’ is the imaginary part of material permittivity, and ***E*** is the electric field of the medium. Since the distribution of nanoparticles in the working base fluid is random, the incidence and polarization direction of the electromagnetic wave are changed here to calculate the average of the three components (i.e., *x*-incidence with *z*-polarization, *x*-incidence with *y*-polarization and *z*-incidence with *x*-polarization) [37].

## 3. Results and Discussions

### 3.1. Absorption and Scattering Efficiencies and Resonance Mode of Plasma Dimer

**Figure 2:**
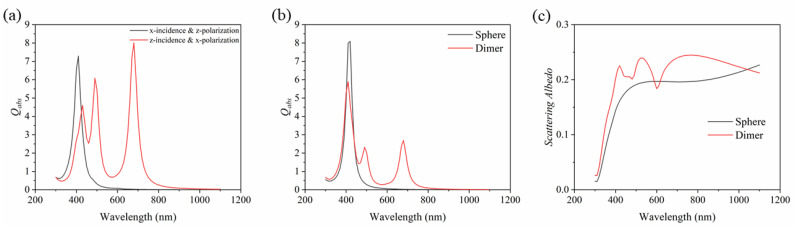
(**a**) Absorption efficiency of dimer in two directions, (**b**,**c**) comparison of absorption efficiency and scattering albedo between plasma dimer and ordinary single Ag sphere nanoparticle (curves are averaged over the three directions, and the three directions are *x*-incidence with *z*-polarization, *z*-incidence with *x*-polarization and *x*-incidence with *y*-polarization).

In this section, we mainly discuss the tangent particles in the dimer. Due to the symmetry of the structure, the case of *x*-incidence with *z*-polarization is exactly the same as that of *x*-incidence with *y*-polarization. Therefore, only the two cases of *x*-incidence with *z*-polarization and *z*-incidence with *x*-polarization are considered here. Figure 2a shows the absorption efficiency of the plasma dimer nanoparticle in two directions. For *x*-incidence with *z*-polarization, the excited resonance peak in the dimer appears at 410 nm, and the absorption efficiency is 7.3. For *z*-incidence with *x*-polarization, three resonance peaks are excited in the dimer. The first is located at 430 nm with an absorption efficiency of 4.6; the second resonance peak is around 490 nm, and the absorption efficiency is 6.1; the third resonance peak is located at 680 nm, and the absorption efficiency is 8.0; the three absorption peaks are all clear. Figure 2b shows the comparison of the average absorption efficiency in the three directions between the proposed Ag plasma dimer nanoparticle and an ordinary Ag sphere nanoparticle. It can be observed that a sharp absorption peak occurred in the Ag sphere nanoparticle near 420 nm. For the dimer nanoparticle, there is also an absorption peak at the same wavelength. In addition, compared with the Ag sphere nanoparticle, one can see that more absorption peaks can be excited in the dimer. This trend means that Ag plasma dimer nanoparticles have a broader absorption range and stronger absorption intensity, which is more conducive to absorbing solar radiation. The comparison of the scattering albedo between the plasma dimer and the Ag sphere nanoparticle is shown in Figure 2c. The scattering albedo here is the ratio of scattering efficiency to extinction efficiency, and the extinction efficiency is equal to the sum of absorption efficiency and scattering efficiency [46]. Compared with the ordinary Ag sphere nanoparticle, the scattering albedo of the dimer is slightly higher. Although the increased scattering albedo here causes the photons to be scattered out of the collector, which may reduce the absorption of solar energy, the effect is minimal because the increase is small. Moreover, light scattering can increase the optical path length of photons, which is beneficial [47]. It is worth noting that nonclassical effects might also appear between the gap of the two closely located nanospheres, which can affect sunlight scattering and absorption [48]. However, this discussion is out of the scope of this study.

**Figure 3:**
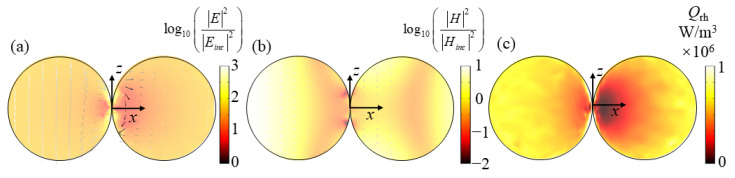
(**a**–**c**) show the electric/magnetic field and resistive heat distributions of dimer at *λ* = 410 nm for *x*-incidence with *z*-polarization, respectively.

Next, we analyze the resonance modes of all absorption peaks in Figure 2a through the obtained electromagnetic field. Figure 3a,b show the electric and magnetic fields at the absorption peak *λ* = 410 nm when the dimer is at *x*-incidence with *z*-polarization, respectively. It can be seen from Figure 3a that the electric field at the corner of the combination of the two spheres nanoparticle in the dimer is significantly enhanced, and the electric field intensity is approximately symmetrically distributed along the *x*-axis. For the magnetic field in Figure 3b, the magnetic fields in the leftmost and rightmost parts and the middle right part of the dimer are enhanced. The electric vector direction is consistent with the direction of the incident electric field and conforms to the characteristics of electric resonance [49]. The enhanced electric field is attributed to the LSPR effect, which has been verified to be able to occur in the nanogaps between closely located metallic nanoparticles [50,51]. This LSPR phenomenon can be understood as the coupling and hybrid of the plasmons induced by single nanoparticles in the dimer. Figure 3c shows the resistance heat distribution at the resonance peak. It can be seen that the LSPR at the junction not only increases the electric field but also enhances the resistance heat distribution. These heat losses will then enter the surrounding liquid from the particles to realize the conversion of the electromagnetic energy of sunlight to the thermal energy in the liquid, thereby improving the energy conversion efficiency of the specific photothermal effect.

**Figure 4:**
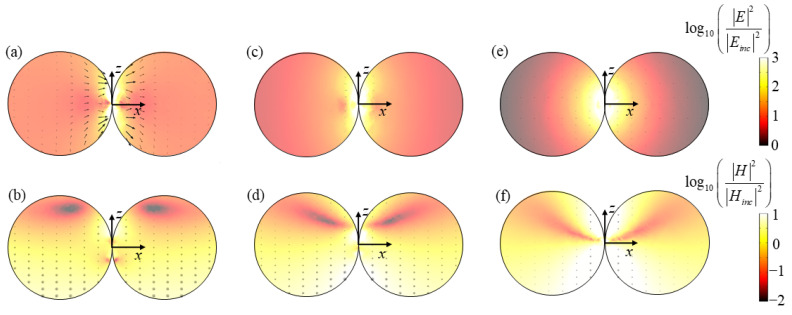
The electric/magnetic field distribution of dimer at (**a**,**b**) *λ* = 430 nm, (**c**,**d**) *λ* = 490 nm and (**e**,**f**) *λ* = 680 nm for *z*-incidence with *x*-polarization.

Here, the case of *z*-incidence with *x*-polarization is discussed, which corresponds to three absorption peaks of 430 nm, 490 nm and 680 nm. Figure 4a,b illustrate the electric and magnetic fields at the peak of *λ* = 430 nm. In Figure 4a, it is clear that the electric field strength at the junction of the dimer is greatly enhanced, and the magnetic field strength decreases gradually along with the incidence direction. In the positive half of the *z*-axis of the dimer, there are two clear attenuation regions of magnetic field strength. Moreover, the direction of the magnetic field is symmetrically distributed with respect to the *z*-axis in Figure 4b. Figure 4c,d present the electric and magnetic fields at the peak of λ = 490 nm. Similarly, it can be clearly seen from Figure 4c that the electric field strength at the junction of the dimer is greatly enhanced. The direction of the electric vector is consistent with the direction of the incident electric field. At the junction indicated in Figure 4d, the magnetic fields at the lower part of the negative z-axis and the upper part of the positive z-axis of the dimer have been greatly enhanced. Figure 4e,f show the electric and magnetic fields at the peak of λ = 680 nm. It can be observed from Figure 4e that the electric field strength at the junction of the dimer is also greatly improved. As for the magnetic field in Figure 4f, the magnetic field intensity gradually attenuates along the incident direction, and the magnetic field intensity is symmetrically distributed along the *z*-axis.

**Figure 5:**
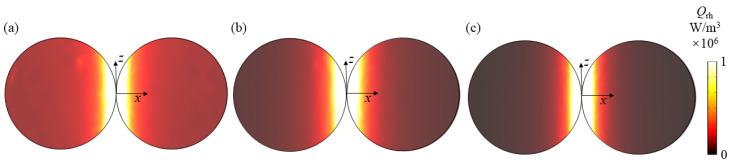
The resistive heat distribution of dimer at (**a**) *λ* = 430 nm, (**b**) *λ* = 490 nm and (**c**) *λ* = 680 nm for *z*-incidence with *x*-polarization.

Similarly, the increase in the electric field intensity at the above three resonance peaks all occur at the junction, and the direction of the electric vector is consistent with the direction of the incident electric field, which conforms to the electric resonance characteristics [49]. Moreover, the enhanced electric field is attributed to the LSPR effect, which has been verified to be able to occur in the nanogaps between closely located metallic nanoparticles [50,51]. This LSPR phenomenon can be understood as the coupling and hybrid of the plasmons induced by single nanoparticles in the dimer. Therefore, the resonance modes at the above three resonance peaks are all considered to be LSPR. In addition, Figure 5 shows the resistive heat distribution at the resonance peak. It can be seen that the resistive heat distribution at the junction is greatly enhanced due to the effect of LSPR.

By analyzing the resonance mode of all absorption peaks excited by light in the dimer, it is shown that the absorption peaks excited by the dimer are attributed to electrical resonance (i.e., LSPR). Compared with the ordinary Ag nanoparticle, the dimer reduces the absorption sharp point near *λ* = 410 nm, widens the absorption range and excites the absorption peak at 490 nm and 680 nm, which is very beneficial to improve the absorption of the solar visible light region.

### 3.2. Effects of Geometric Parameters and Embedding during Synthesis

**Figure 6:**
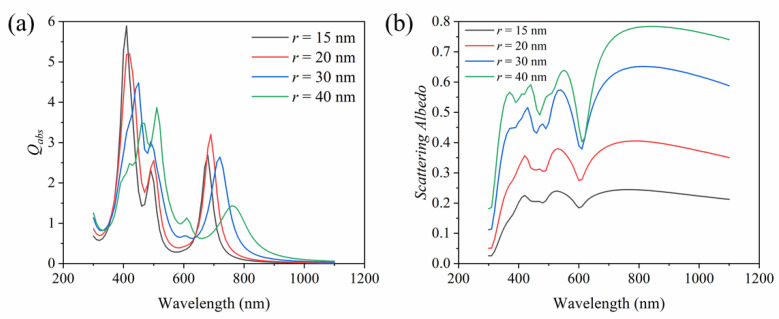
Effects of geometrical size on (**a**) absorption efficiency and (**b**) scattering albedo of dimer nanoparticle (average of three directions).

The structure and size of nanoparticles have an important impact on the absorption properties of nanoparticles [39]. In order to apply the proposed plasma dimer nanoparticle in DASC, the geometrical size and the effect of embedding during synthesis are considered. As shown in Figure 6, the effect of the radius *r* of the Ag sphere on the absorption efficiency and scattering albedo of the dimer nanoparticle is discussed. Figure 6a shows the absorption efficiency of the dimer when the radius of the Ag sphere is different. The difference of *r* causes a significant change in the absorption peak. With the increase in radius *r*, the absorption peak near 410 nm decreases gradually, but the absorption peak becomes broader, which is conducive to the absorption of solar energy. Meanwhile, the absorption peak is redshifted. The absorption peak near 490 nm is enhanced with the increase in *r*. Compared to the peak value of 2.7 at *r* = 15 nm with *λ* = 680 nm, the absorption peak of *r* = 20 nm with *λ* = 690 nm has a higher value of 3.2. Moreover, the absorption peak redshifts and gradually decreases when *r* increases again, but the absorption peak is broader, which is beneficial to the absorption of solar energy. The above results show that the absorption band and absorption peak can be adjusted effectively by adjusting the geometric parameters of the nanoparticle so that it is beneficial to the solar absorption. In addition to absorption, the scattering of nanoparticles also has an important impact on the performance of the solar collector [52]. This is because scattering causes photons to be scattered away from the collector; in other words, it reduces the absorption of solar energy. However, from another perspective, this is not entirely harmful, because light scattering increases the optical length of photons [47]. Figure 6b shows the scattering albedo of the dimer when *r* is different, and the scattering albedo of the dimer increases with the increase in *r*, which indicates that the tuning of the scattering efficiency is possible. Therefore, in practical applications, the scattering can be achieved within a reasonable range by optimizing the geometric parameters, which further contributes to the absorption of solar energy.

**Figure 7:**
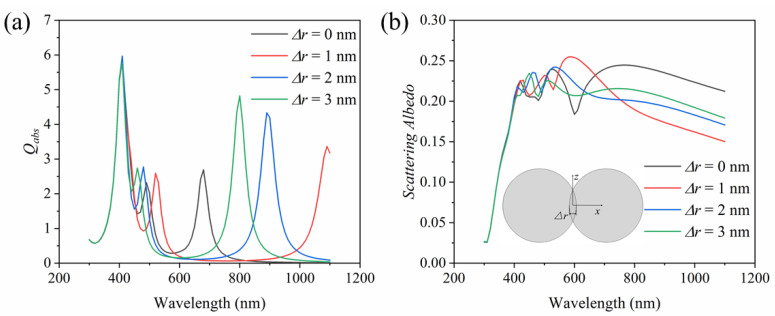
Effects of embedding on (**a**) absorption efficiency and (**b**) scattering albedo of dimer nanoparticle during synthesis (mean of three directions).

In the process of synthesis, two spheres may deviate from being completely tangent and embedded. Figure 7 presents the effect when the two spheres are embedded. As can be seen from Figure 7a, the first absorption peak hardly changes, which is caused by the main LSPR around the sphere. Without considering Δ*r* = 0, with the increase in Δ*r*, the second and third absorption peaks are blueshifted, which is caused by the gap resonance between the two spheres as the gap decreases with the increase in the embedding depth Δ*r* [35]. However, compared with Δ*r* = 0, the second absorption peak is blueshifted or redshifted after the embedding between the two spheres, and all of the third absorption peaks are redshifted. Figure 7b shows the scattering albedo of the dimer with different embedding depth. With the increase in embedding depth, the intensity of the scattering albedo does not significantly change. In conclusion, the embedding produced in the synthesis process has a favorable effect on the absorption efficiency of the plasma dimer, and the occurrence of embedding will lead to redshifted or blueshifted, which will further improve the absorption of solar energy.

**Figure 8:**
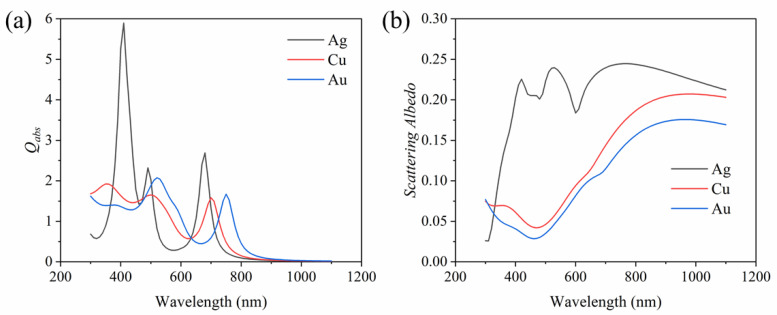
Effects of different metal materials on (**a**) absorption efficiency and (**b**) scattering albedo of dimer nanoparticle.

In addition, the material of nanoparticles can also have an important impact on their optical properties. Therefore, we investigate the absorption efficiency and scattering albedo of the plasma dimers based on several common materials (i.e., Cu and Au). The absorption efficiency of the dimer nanoparticle with different metal materials is shown in Figure 8a. Clearly, the materials of the plasma dimer nanoparticle have a great influence on the absorption efficiency. The absorption efficiency of the Ag dimer is described above. For the Cu dimer nanoparticle, there are three absorption peaks at the wavelengths of 350 nm, 500 nm and 700 nm. For the Au dimer nanoparticle, a main peak with an absorption efficiency of 2.1 appears at the wavelength of 520 nm, and another peak occurs at around 750 nm. Figure 8b compares the scattering albedo of the dimer with the three different materials. It can be clearly seen that with the increase in wavelength, the scattering of the Ag dimer nanoparticle at first increases rapidly and then remains at a relatively large value. While the scattering of the Cu dimer nanoparticle drops down at first and then increases gradually and reaches 0.2. For the Au dimer nanoparticle, the trend of the scattering is similar to that of the Cu dimer nanoparticle, but the scattering of the Cu dimer is higher. In summary, adjusting the combination of different metal nanoparticles to adjust the energy window required for solar energy absorption has the potential to improve solar energy absorption and requires further development.

### 3.3. Trimers and Their Special Structures

**Figure 9:**
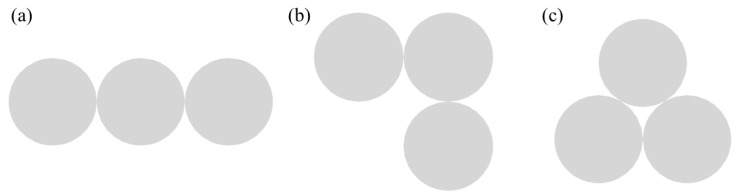
Ag plasma trimers with three special structures: (**a**) column, (**b**) right angle, (**c**) triangle.

**Figure 10:**
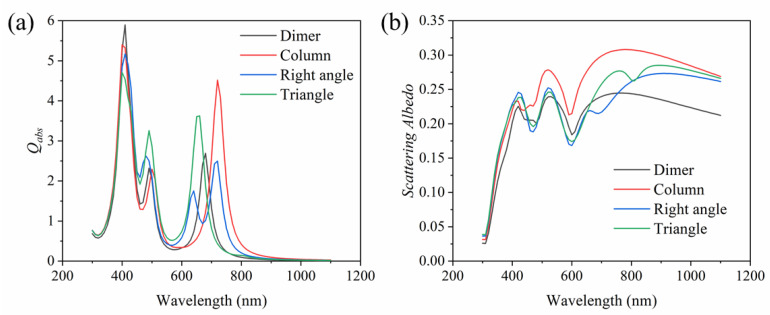
Comparison of (**a**) absorption efficiency and (**b**) scattering albedo of plasma dimer and three Ag plasma trimers with special structures.

In addition, we studied the synthesis of plasma trimers by three Ag spheres. Figure 9a–c are schematic diagrams of the column structure, right angle structure and triangle structure synthesized by three Ag spheres, respectively. Figure 10 compares the absorption efficiency and scattering albedo (average value of the three directions) between the dimer and these three special structure trimers. It can be clearly seen from Figure 10a that the absorption efficiency of trimers with different structures is different. Similarly, the three structures have an absorption peak near 400 nm, but they are all lower than the absorption peak of the dimer. Moreover, it is found that three absorption peaks occur at the wavelength of 400 nm, 500 nm and 720 nm for the column structure, and the absorption efficiency is 5.4, 2.3 and 4.5, respectively. The absorption peaks of the triangle structure are around 400 nm, 490 nm and 660 nm, and the ratio of absorption efficiency is 4.7, 3.3 and 3.6, respectively. The right angle structure trimer has four absorption peaks, and the main peak with an absorption efficiency of 5.2 is located near 410 nm. The other three absorption peaks are located near 480 nm, 640 nm and 720 nm, and the absorption efficiency fractions are 2.6, 1.7 and 2.5, respectively. Compared with the dimer, the column and triangle structure trimers have higher absorption peaks except for the main peak. The right angle structure trimer has one more absorption peak than the dimer, which is more favorable for the absorption of solar energy. Moreover, compared with the dimer, the trimer can better enhance the absorption at the resonant wavelength. This is because all of the gaps of the trimer have strong resonances, and the resonances at the gaps are superimposed on each other, resulting in an enhanced electric field of the entire particle area. Figure 10b shows the comparison of the scattering albedo of the dimer and the three different structures. The scattering of the trimer is slightly larger than that of the dimer on the whole. Moreover, the overall scattering of the column structure is relatively stronger, while the scattering albedo of the right angle and triangle structures is very similar.

**Figure 11:**
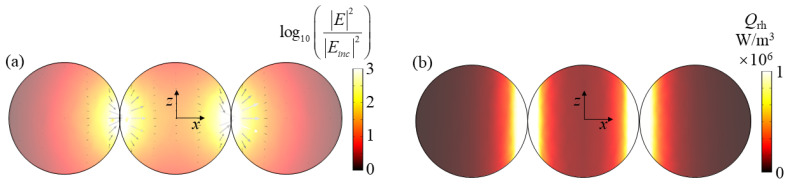
The (**a**) electric field and (**b**) resistive heat distributions of trimer at *λ* = 720 nm with *z*-incidence and *x*-polarization.

Figure 11 shows the electric field and resistive heat distributions of the plasma trimer (column structure) at the wavelength of 720 nm when the applied plane wave is *z*-incidence with *x*-polarization. As shown in Figure 11a, it is clear that due to the strong resonance at the gap, the electric field strength at the Ag ball joint is greatly increased. The direction of the electric vector is consistent in the direction of polarization, which indicates the properties of LSPR. Moreover, the enhancement of electric field intensity is concentrated at the junction. In summary, this electric resonance mode is still considered LSPR. In addition, as shown in Figure 11b, due to LSPR, the resistive heat distribution at the junction of the Ag balls is greatly enhanced.

**Figure 12:**
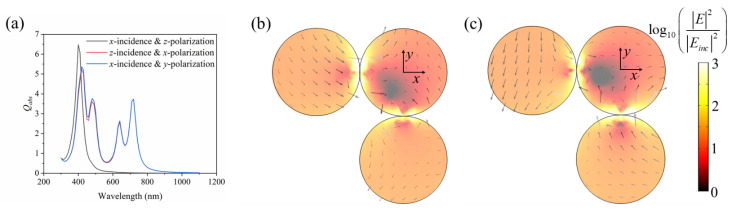
(**a**) Absorption efficiency in three directions of a right-angled trimer and the electric field of (**b**) *z*-incidence with *x*-polarization and (**c**) *x*-incidence with *y*-polarization at λ = 420 nm.

Interestingly, in the three special trimers, the right angle structure excited more absorption peaks. This is because the column and triangle structures are centrosymmetric, while the right angle structure is not. The case of the right angle trimer is discussed in detail below. Figure 12a shows the comparison of the absorption efficiency of the trimer with the right angle structure in three directions. Compared with the other two structures, the right angle structure excited more absorption peaks in *z*-incidence with *x*-polarization and *x*-incidence with *y*-polarization than the other two structures. Because of the asymmetry of the structure, the absorption peak depends on the direction and polarization of the incidence wave. For *x*-incidence with *z*-polarization, it is consistent with the previous statement. Due to the electrical resonance, the formant occurs near 400 nm. For both *x*-incidence with *y*-polarization and *z*-incidence with *x*-polarization, four absorption peaks can be found at the wavelength of 420 nm, 480 nm, 640 nm and 720 nm. The reason for this is that the polarization of the right angle structure in the *x* direction is similar to that in the *y* direction. Figure 12b,c show the electric field distribution at *λ* = 420 nm in *z*-incidence with *x*-polarization and *x*-incidence with *y*-polarization, respectively. It is clear that the electric field at the joint of the Ag sphere is greatly enhanced, resulting from the interstitial resonance between LSPR and particles. The electric field at the absorption peak excited by the right angle structure is greatly enhanced at the joint of two Ag spheres, and its resonance mode is also attributed to the electrical resonance (LSPR), which will not be described here. In conclusion, the plasma trimer has great potential for solar energy absorption.

The successful synthesis of linear and triangular Au oligomers has been reported [53,54]. By manipulation of the synthesis procedure and conditions, such as surfactants, the number of particles in a cluster and the embedding depth can be controlled. To avoid oxidation and stability of the nanosphere particles, researchers have also proposed different approaches, such as adjusting the pH value and adding a SiO_2_ shell over the Au or Ag nanosphere cores [55]. The multiple absorption peaks caused by different nanoparticles can promote light-to-heat conversion. However, in the application, the cost of various particles must be considered to comprehensively determine which particles to use. The synthesis of the proposed nanodimers is an important direction for future study. In practical applications, it is necessary to mix particles with different absorption peaks to achieve the purpose of broadband absorption.

## 4. Conclusions

In this study, a plasma dimer Ag nanoparticle composed of two Ag spheres of the same size is proposed for solar absorption. The optical properties of the plasma dimer nanoparticle are studied. Several absorption peaks are observed under the incident plane wave. The electromagnetic field analysis of the dimer shows that these absorption peaks are excited in the dimer due to electric resonance (LSPR). Compared with a single Ag nanoparticle, the plasma dimer nanoparticle has more absorption peaks, which can broaden solar energy absorption. The analysis of the size of the nanoparticle shows that the absorption band and the absorption peak of the nanoparticle can be adjusted effectively by adjusting the geometric parameters of the nanoparticle so that it is conducive to the broadband absorption of solar energy. The effect of embedding between Ag spheres during dimer synthesis is also considered, which leads to more absorption peaks and broader solar energy absorption and further improves the absorption of solar energy. In addition, the synthesis of a trimer with three Ag spheres of the same size is investigated. Through the analysis of three special structures of trimers, it is found that the plasma trimer has great potential to improve the absorption of solar energy. The above work shows that the proposed plasma dimer nanoparticle is of great significance to improve the absorption of solar energy and has broad prospects for the application of solar thermal conversion technology.

## Data Availability

Data underlying the results presented in this paper are not publicly available at this time but may be obtained from the authors upon reasonable request.
